# Complications after Prostate Cancer Treatment: Pathophysiology and Repair of Post-Radiation Urethral Stricture Disease

**DOI:** 10.3390/jcm12123950

**Published:** 2023-06-09

**Authors:** Joshua Sterling, Syed N. Rahman, Ajin Varghese, Javier C. Angulo, Dmitriy Nikolavsky

**Affiliations:** 1 Yale School of Medicine, 20 York Street, New Haven, CT 06511, USA; joshua.sterling@yale.edu (J.S.); syed.rahman@yale.edu (S.N.R.); 2 New York College of Osteopathic Medicine, 8000 Old Westbury, Glen Head, NY 11545, USA; avargh04@nyit.edu; 3 Faculty of Biomedical Sciences, Universidad Europea, 28905 Madrid, Spain; javier.angulo@universidadeuropea.es; 4 SUNY Upstate Medical College, Syracuse, NY 13210, USA

**Keywords:** radiation injury, urethral stricture, reconstructive urology, wound healing, cancer survivorship

## Abstract

Radiation therapy (RT) in the management of pelvic cancers remains a clinical challenge to urologists given the sequelae of urethral stricture disease secondary to fibrosis and vascular insults. The objective of this review is to understand the physiology of radiation-induced stricture disease and to educate urologists in clinical practice regarding future prospective options clinicians have to deal with this condition. The management of post-radiation urethral stricture consists of conservative, endoscopic, and primary reconstructive options. Endoscopic approaches remain an option, but with limited long-term success. Despite concerns with graft take, reconstructive options such as urethroplasties in this population with buccal grafts have shown long-term success rates ranging from 70 to 100%. Robotic reconstruction is augmenting previous options with faster recovery times. Radiation-induced stricture disease is challenging with multiple interventions available, but with successful outcomes demonstrated in various cohorts including urethroplasties with buccal grafts and robotic reconstruction.

## 1. Introduction

About 192,000 cases of prostate cancer are diagnosed annually, with patients undergoing treatments such as prostatectomy, external beam radiation therapy (EBRT), and brachytherapy (BT) [1]. The Surveillance, Epidemiology, and End Results (SEER) database found that 13% of patients with prostate cancer opt for BT, 25% of patients opt for EBRT, and 38% of patients opt for prostatectomy [2]. As popular as these treatment modalities are, they present with significant side effects that can profoundly affect the quality of life of patients. Radical prostatectomy can result in urinary incontinence in 5–72% of patients [3,4]. Patients can elect to undergo different procedures to address this complaint, with the most frequent option being urethral slings (47.5%), artificial urinary sphincter (AUS) (35.3%), and the injection of bulking agents (17.2%) [3].

RT use in managing pelvic cancer has been shown to lead to multiple sequelae including radiation-induced stricture disease. This has created a complex urologic issue for urologists, notably reconstructive urologists, due to the increased rates of recurrence and complications after treatment of stricture disease, among other sequelae, after the application of ionizing radiation. The CaPSURE (Cancer of the Prostate Strategic Urologic Research Endeavor) database analysis approximates that the likelihood of radiation-induced stricture is around 1.8% for BT and 1.7% for EBRT, while EBRT + BT combination therapy led to an overall likelihood of 5.2% [5].

This review aims to examine the current literature regarding urethral stricture induced by radiation in male patients, explicitly focusing on the effects radiation can have on histology, biochemistry, and biology in tissue, as well as pathogenesis before reviewing the literature regarding the management of radiation-induced urethral strictures.

## 2. Prevalence

Pelvic RT administered for rectal cancer can impact the bladder and urethra due to the short distance between these organs. Urinary adverse events (AE) are to be expected; however, there is a dearth of studies following potential urologic sequela [6]. Studies that tracked urinary AE revealed that about 4% of patients experienced severe AE [7]. When examining patients diagnosed with prostate cancer who solely received EBRT, a study performed by David et al. revealed a 28.4% 10-year cumulative incidence rate of hospital admissions due to urinary AE [8]. The absence of studies presents a potential future area of exploration and collaboration among diverse disciplines and specialties.

Muise et al. conducted a review of a cohort of 67,527 patients diagnosed with prostate cancer; 72.62% received RT, 24.86% underwent a radical prostatectomy (RP), 4.94% underwent a RP followed by RT, and 0.46% received RT followed by RP. Within five years of completing treatment, 8.44% of those treated with RT developed urethral strictures, compared to the 5.35% diagnosed with strictures after RP [9]. When both modalities were used, the rates of urethral stricture were higher, with 11.22% of patients experiencing stenosis after salvage RT and 19.34% after salvage RP [9].

Thirty eight percent of patients with a new diagnosis of PCa choose RT as a treatment modality, and BT and EBRT are the most common options [2]. Radiation sequelae present an issue that continues to occur for urologists when following up with patients who underwent RT. In Australia, a tertiary referral urology department discovered that 2.8% of their admissions were due to radiation-induced disease; 7.2% of admissions stemmed from the emergency room [10]. As with most sequelae of RT, urethral stricture rates depend on the radiation dosage received. BT can induce stenosis at a rate ranging from 1.7% to almost 32% for patients undergoing high-dose BT [5,11,12,13,14]. EBRT alone can result in a 1.1–4.5% risk of stricture formation, which is magnified when combined with BT [5,12,15,16]. A systematic review conducted by Awad et al., involving over 16,000 patients who underwent RT, revealed a urethral stricture incidence rate of 3.9%, and a pooled estimate period prevalence of 2.2% four years after the procedure. In contrast, the pooled prevalence of combined EBRT + BT was found to be 4.9% [6,12]. As the length of follow up increases, so does the risk of developing strictures. For reported strictures occurring after BT, 90–100% of them are in the bulbomembranous urethra and happen a median of 2.2–2.4 years after the completion of radiotherapy [12,13,14].

## 3. Stricture Etiology/Physiology

When the urethral lumen is subjected to repetitive damage, an atypical narrowing is formed due to these damages causing structural alterations to the supporting spongiosum and connective tissue underlying the urethral lumen [17,18]. As a result of these alterations, urine can extravasate into the neighboring spongiosum and trigger fibrotic changes. This process leads to the formation of a plaque which is classified as a stricture if it is circumferential [19]. The fibrosis present in urethral strictures demonstrates unique properties when compared to fibrosis in other areas of the body, such as a protracted healing process and involvement of much of the periurethral tissue [20]. In a study by Hofer et al., it was found that the parallels between urethral healing and dermal wound healing consisted of acute inflammatory, proliferative, and remodeling phases. These phases exhibited a 50% longer duration with cellular infiltration and cytokine changes that went beyond the injury site and affected a significant portion of periurethral tissue [20].

Important distinctions between urethral remodeling and dermal remodeling include differences in the length of the proliferative phase, which peaks at day 5 in dermal remodeling, while in urethral remodeling, students have demonstrated evidence of remodeling on day 10 [20].

Evidence from animal studies suggests strictures result from an increase in collagen and a decrease in smooth muscle content. This leads to compact, fibrotic, and poorly compliant tissue, although it is important to note that these findings have not been established in human studies [21]. Human studies have instead shown a decrease in the ratio of Type I collagen to Type III when comparing normal urethral spongiosum to urethral strictures [22]. Typical urethral spongiosum is composed of 75% Type I collagen and 25% Type III, compared to 16% Type I collagen and 84% Type III in the spongiosum of urethral strictures [22]. Accompanying these modifications are a reduction in smooth muscle and neuronal nitric oxide and an elevation in intrinsic nitric oxide [23]. The decline in neuronal nitric oxide, coupled with the damage to the cavernosal nerves, has been associated with alterations in the corpora cavernosa characterized by an upsurge in fibrosis and a deterioration of smooth muscle [23].

Stricture formation may also be influenced by modifications in the architecture of the extracellular matrix. Da-Silva et al. analyzed extracellular matrix proteins from the bulbar urethral strictures of 10 patients who were treated with excision and end-to-end anastomosis and compared them to the extracellular matrix proteins present in bulbar urethras from fresh cadavers. The findings revealed that hyaluronic acid was 50% lower and that dermatan sulfate was 68% higher within the extracellular matrix of urethral strictures compared to that of healthy urethras [24]. Stricture patients also had a significantly lower (*p* < 0.05) mean total concentration of glycosaminoglycans (GAG) when compared to healthy controls [24].

Additionally, these concepts of the mechanisms of stricture formation in the urethra are not uniform across the urethra. The urethra is not homogeneous and the different histologic properties of each section of the urethra change the etiology and mechanism of stricture formation. The navicular fossa, also called the glandular urethra, consists of stratified squamous epithelium and a layer of connective tissue encompassed by corpus spongiosum [17,25]. The anterior urethra is composed of a pseudostratified columnar epithelium that rests on a basement membrane. Below that basement membrane are the vascular sinusoids of the corpus spongiosum, housed by a layer of connective tissue [17,25]. The membranous urethra traverses through the external sphincter and perineal membrane. Around this point, the pseudostratified columnar epithelial cells begin to transition into urothelium, and skeletal muscle appears at the level of the external sphincter [25]. The prostatic urethra features urothelial cells surrounded by glandular and stromal tissue of the prostate’s transition zone [25]. Finally, the pre-prostatic urethra is entirely within the bladder wall and composed solely of urothelium [25].

Anterior urethral strictures typically arise due to trauma or infection and exhibit a more prominent spongiofibrosis. Posterior stenosis is commonly caused by iatrogenic injuries, such as RT, with a fibrotic plaque that is rarely circumferential [18].

## 4. Radiation-Induced Changes in Urologic Tissue

RT causes unavoidable radiation injury to normal tissues, resulting in acute and late effects on parenchyma, stroma, and vascular structures. Effects that manifest within hours of exposure are characterized as acute changes and can consist of an increase in vascular permeability, lymphocyte adhesion and infiltration, and endothelial cell edema [26]. Effects manifesting months to years later are characterized as late changes and result from the reduction in stem cells or progenitor cells. Late effects include organ dysfunction, fibrosis, and necrosis [26].

Following RT, the urothelium exhibits parenchymal and epithelial changes such as cellular atypia, neoplasia, dysplasia, metaplasia, necrosis, and atrophy. One of the most notable and consistent delayed effects of RT is atrophy [27]. Delayed necrosis present in the acute phase is usually secondary to ischemia, leading to fissures in the epithelial lining. Fibrosis, the presence of fibrinous exudate, and atypical fibroblasts are stromal lesions that are commonly visualized in the lower urinary tract.

Radiation-induced fibrosis and vascular insufficiency present as delayed effects. The extent of these sequelae is dependent on the surrounding tissues that are impacted and, in the case of blood vessels, is proportional to the size of the vessels impacted. Damage to small capillaries typically results in obliteration, as they are most radiosensitive leading to endothelial swelling and increased permeability, while damage to medium-sized vessels results in fibrinoid necrosis and thrombosis [28]. Damage to large vessels is rare.

Radiation-induced endothelial apoptosis plays a prominent role in the series of acute vascular changes. Late-stage radiation changes produce vascular effects involving thickening of the basement membrane, scarring of surrounding tissues alongside the development of telangiectasias, and a decline in clonogenic capacity, which presumably contributes to the late-stage radiation response to typical parenchyma [29].

## 5. Pathways of Radiation-Induced Endothelial Cell Death

The behavior of endothelial cells when exposed to radiation has been extensively studied and understood. The macroscopic changes observed in acute radiation toxicity occur when irradiated endothelial cells undergo structural changes and produce a range of growth factors, chemoattractants, and biomarkers [26].

The prior literature has investigated different pathways involved in radiation-induced stricture formation; however, they are predominantly influenced by damage to the membrane and are mediated by ceramide production and acid sphingomyelinases (ASMases). Most studies exploring this pathway utilized single doses of radiation ranging from 10 to 20 Gy [6,26]. Thus, the specific function this pathway may have in the formation of urethral strictures at clinically relevant doses of radiation remains unclear [26]. Studies have shown that ceramide, which is generated from sphingomyelin, exerts a substantial impact on the radiation-induced apoptosis pathway. Ceramide can function as a secondary messenger and can also be transformed into a structural or effector molecule. Ceramide creation occurs through the action of a neutral or ASMase, while ceramide synthase mediates the de novo synthesis of ceramide. Within endothelial cells, the increase in intracellular ceramide levels following radiation exposure has been associated with the induction of ASMase resulting from subsequent membrane damage [6,30]. There are three ways for ceramide to be released from membrane-bound sphingosine: ASMase activation, cell membrane damage from radiation, or the binding of death-receptor ligands [26].

Ceramide activates the ceramide-activated protein kinase (CAPK) and the ceramide-activated phosphatase, leading to apoptosis through the activation of the MAPK8 pathway, the mitochondrial pathway, and the death receptor pathway [26,31,32]. The inhibition of protein kinase C (PKC) by ceramide-activated phosphatase is a critical step, given that PKC participates in antiapoptotic signaling and can impede sphingomyelin hydrolysis, thus obstructing the release of ceramide from cellular membranes [33,34,35]. In addition to these targets, the essential pathway ceramide target is the RAC1/MEKK1 pathway, which would otherwise activate MAPK8 amongst other steps. Once MAPK8 is activated, the effector caspases (1, 3, and 6) and autocrine stimulation of the death receptor pathway through tumor necrosis factor (TNF) result in apoptosis [26,36].

Caspase 9 functions as an alternative activation pathway of caspases for apoptosis through mitochondrial proteins. This process is initiated by ceramides, as well. BCL-2-associated protein x (BAX) and BCL-2 antagonist of cell death protein (BAD) are proapoptotic proteins that promote apoptosis when CAPK is induced by ceramide [37]. When BAD binds to antiapoptotic proteins BCL2 and BCL2L1, cytochrome C is released and then caspase 9 is activated, which stops the suppression of apoptosis which is controlled by BAX [38,39]. In order for cell death to be instigated, cell death substrates need to be degraded. This is achieved through caspase 9 since it is able to sever and induce the downstream effector caspases, which results in the initiation of apoptosis [6,40].

Ceramide can be released through cell death or TNF receptor activation. This release leads to a direct apoptotic pathway using different adapter protein complexes including the TNFαR-associated death domain and the Fas-associated death domain [26]. The aforementioned domains start the induction of cytoplasmic promoters of cell death such as procaspase 8, which cleaves and activates effector caspases.

When cell death or TNF receptor activation occurs, ceramide is released and a direct apoptotic pathway using different adapter protein complexes is initiated, some of them being the Fas-associated death domain and the TNFαR-associated death domain [26]. These domains start the activation of cytoplasmic promoters of apoptosis such as procaspase 8, which cleaves and activates the effector caspases.

Ceramide can also be released from radiation-induced DNA damage since the ceramide synthase can be activated by DNA double-strand breaks. This pathway would need de novo protein synthesis, which would result in slower kinetics over a longer period of time as a proapoptotic mechanism compared to the sudden release of ceramide once ASMase is activated [26]. DNA damage from radiation can trigger p53-dependent processes and contribute to cell death since p53 affects the transactivation of genes related to pro- and antiapoptotic cascades, as well as the maintenance of cell cycle progression. BCL2 and BAX proteins are directly controlled by p53, which can determine whether or not the proteins are activated. p53 is also responsible for inducing apoptosis through upregulating the death receptor–ligand system [41,42].

The balance between pro- and anti-apoptotic signaling cascades and radiation dose determines the amount of endothelial cell apoptosis. For example, ionizing radiation does not lead to endothelial apoptosis directly from the mechanisms listed above, but it also activates anti-apoptotic pathways. A study by Tan et al. revealed that the induction of protein kinase B/AKT (PKB/AKT) signaling determines the viability of endothelial cells that have undergone a single dose of radiation (3 Gy) [6,43]. When PKB/AKT is induced, glycogen synthase kinase-3beta (GSK3beta) is suppressed. This pathway functions to prevent apoptosis since GSK3beta plays a part in decreasing endothelial cell viability [43].

Toulany et al. found that radiation-induced PKB/AKT signaling is a part of cell survival after the application of ionizing radiation through the stimulation of DNA double-stranded break repair through the induction of DNA-PK [44]. DNA-PK is an important enzyme involved in the nonhomologous end-joining repair mechanism [44]. Overall, there is a dose-dependent relationship in radiation-induced stricture formation that heavily relies on balancing the pro- and anti-apoptotic mechanisms listed above.

## 6. Cellular and Extracellular Components of Fibrosis

Fibrotic tissue changes are the result of the decreased decomposition and increased production of extracellular matrix (ECM) proteins, especially collagen, which can be caused by radiation exposure. The premature terminal differentiation of potentially mitotic progenitor fibroblasts into irreversible postmitotic fibrocytes can be activated by ionizing radiation [45,46,47]. Growth factors, tissue-specific collagen, cytokines, and matrix molecules are produced by these differentiated fibrocytes that are the main component of the fibroblast system [46,47,48]. This accumulation of postmitotic fibrocytes may explain why radiation results in an increased synthesis and extracellular deposition of collagen, which is remarkable for post-radiation fibrotic tissue.

Cytokines are important in the mechanisms of radiation-induced injury. TGF-β1 is a cytokine responsible for the proliferation and differentiation of fibroblasts into postmitotic fibrocytes, which secrete collagens and other extracellular matrix proteins. TGF-β1 also controls extracellular matrix homeostasis, which is responsible for the increased production and decreased degradation of extracellular collagen molecules [26,27,28]. The activation of TGF-β1 and plasmin activator inhibitor 1 by radiation exposure plays a significant role in the process of fibrotic tissue changes.

IL-17 is another cytokine that plays an important role in mediating the response of cells to radiation-related damage such as neutrophil recruitment [49,50]. Bessout et al. were able to show that IL-17 creation was increased in CD4 T cells in mice that had undergone colorectal irradiation [51]. Other cytokines that appear after irradiation are cytokines IL-1β and TGF-β [51]. They are involved in Th17 differentiation, a CD4 T cell which secretes IL-17 [51,52]. Since IL-17 is linked to fibrogenesis in conditions of the liver, lungs, and skin, it is speculated that an increase in Th17 could drive the fibrotic changes in irradiated tissue [51,53,54]. Another study by Paun et al. examined lung injuries including pulmonary fibrosis induced by radiation in mice and showed how the linear combination of Th17 and Th1 was a substantial indicator for the extent of pulmonary fibrosis [49]. The levels of IFN-γ and IL-17 together in the bronchoalveolar lavage also correlated significantly with late-stage fibrosis [49].

Excessive accumulation of ECM proteins such as collagen is the most commonly observed component of tissue fibrosis after radiation exposure, mainly through increased synthesis and decreased degradation. The reduced degradation of extracellular collagen that is newly synthesized and deposited can be attributed to the gene expression and production of tissue inhibitors of matrix metalloproteinases (MMP), which is mediated by TGF-β1 [55]. Another mechanism for fibrinolysis and ECM degradation regulation is the plasmin activator (PA) system. Plasmin can break down ECM through proteolytic activity and induction of latent MMPs [56]. The PA system is controlled by a group of PA inhibitors, and plasmin activator inhibitor (PAI-1) is the most significant. TGF-β1, TNF-α, and IL-1 are signaling molecules that can stimulate PAI-1 secretion. Following radiation exposure, the primary factor that appears to induce PAI-1 is the radiation-induced activation of TGF-β1 [26,57]. TGF-β1 plays a role in the homeostasis of ECM proteins, cell growth regulation, radiation-induced fibroblast differentiation and activation, and cellular differentiation. However, the total molecular actions and influence of TGF-β1 has not been fully explained. What can be concluded is the prominence of TGF-β1 in radiation-induced tissue reactions. The extracellular deposition and increased synthesis of interstitial collagen from induced terminally differentiated fibrocytes can be influenced by TGF-β1 or activated through radiation, resulting in the post-radiation connective tissue changes that are labelled as fibrosis.

## 7. Radiation-Induced Histologic Changes

**Figure 1:**
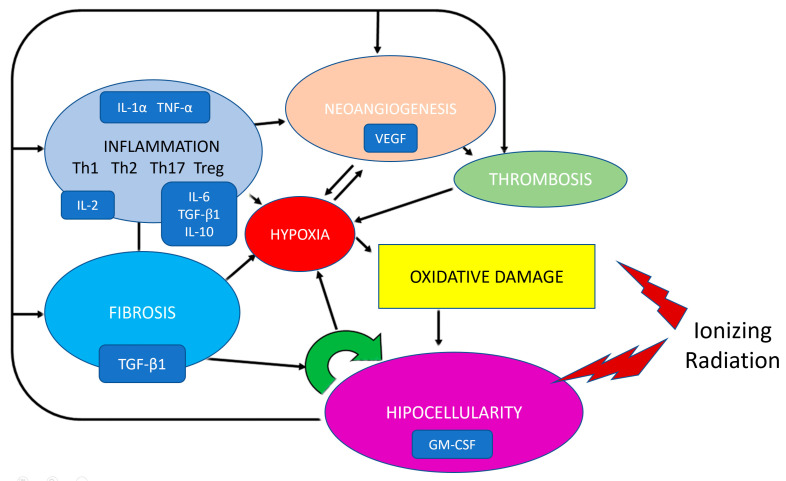
Simplified model of the complex network of interacting processes and signals in the pathogenesis of radio-induced injury.

Radiation causes a variety of histologic alterations including vascular modifications, fibrotic changes, cellular depletions, and inflammatory responses, which manifests progressively as the tissue gets further from the site of radiation exposure (Figure 1; Table 1).

Gallet et al. used a mouse model to combine the histologic modifications alongside the levels of growth factors and cytokines that stimulated these modifications in an attempt to create a histologic scoring system (Table 1). There was a clear relationship found between elevated fibrosis, vascular scoring, and TGF-B1 expression [58]. Levels of stem-cell-mobilizing cytokine GM-CSF, VEGF, and IL-2 were related to the cellular alteration score [58]. While the authors only investigated alterations in muscle and skin, it would be plausible to extrapolate these findings out to other organs.

Pelvic organs and tissue, such as the bladder, may develop radiation cystopathy due to the relatively small spaces in which they are confined. A bladder developing radiation cystopathy may show transient erythema in the first 24 h, progressing to edema, necrosis of basal urothelial cells, urothelial desquamation, ulceration, and finally, hyperemia about 3–6 weeks later [59,60,61]. Chronic radiation changes can cause ulcers, fibrosis, and increased lower urinary tract symptoms (LUTS). Radiation cystitis or persistent hematuria can be caused by the proliferation of telangiectatic vessels [59]. Ureteral stenosis from radiation-induced fibrosis can also occur if radiation effects manifest in the distal ureters. At the molecular level, radiation affects the urothelium in two stages: acute/reversible modifications and late/persistent modifications. Acute insult leads to elevated leukocyte infiltration and, subsequently, LUTS [62]. Radiation injury also increases levels of urothelial intercellular adhesion molecule 1 (ICAM1) and TGF-B, with changes in ICAM1 expression contributing to acute and late-phase changes in the bladder after RT [63,64]. Late/persistent changes tend to result in a loss of umbrella cells and downregulation of uroplakin-III, which exposes the basal layer of the bladder to the caustic components of urine. As stated previously, TGF-B expression is increased, which leads to collagen deposition in the ECM and a permanent reduction in bladder compliance [62]. The prostate gland is commonly a direct target of RT. Damage to adjacent structures has been demonstrated in rat models, with neuronal and vascular damage leading to erectile dysfunction [65,66].

## 8. Pathogenesis of Radiation Strictures

The dynamics of typical cells during and after RT, especially in relation to the manifestation of stricture formation, is patient dependent and, as such, cannot be observed [67,68]. When examining the risk of stricture formation in BT versus EBRT, the ASCENDE-RT trial was able to discover an actuarial incidence rate of 5.2% in patients who underwent EBRT and 18.4% in patients who underwent BT [69,70]. However, combination EBRT + BT was the only risk factor that reliably demonstrated an elevation for the chance of stricture formation [12]. This implies that cumulative radiation dose is the most predictive factor of stricture formation rate. High-dose-rate BT and EBRT resulted in about a 9–21% risk of stricture formation, with conformal and intensity-modulated radiotherapy resulting in a 1.7–4% risk, the lowest possible incidence of stricture [5,11,12,13,14,16,71]. Meta-regression analysis and multivariable regression have not shown a connection among stricture formation and biological equivalent dose, despite the possibility that radiation dose schedules might elevate the chance of stricture formation [11,12,72,73]. The reason for this that inconsistency could occur because the interval of follow up across RT studies, endpoints, and terminology is not standardized. An example of this is that urethral strictures develop over a period of 2.2–3.4 years, and, as such, need long-term follow up to track the progression of formation [5,11]. The Common Terminology Criteria for Adverse Events version 3 mandates that a stricture requires urological intervention in order to be classified as a stricture. Diagnosing a stricture after RT is ultimately left to the provider’s preference.

When RT is administered after surgical intervention, there is a chance for a urethral stricture to develop. This probability does decrease as the amount of time between procedures increases [74]. When RT is applied before surgery, wound healing is disrupted due to damage to fibroblasts and their ability to create collagen, which increases the risk of stricture formation [75]. RT of the prostate is likely to increase the chance of stricture formation, especially in patients undergoing urethral instrumentation. The incidence of urethral stricture formation can also be increased through other factors such as hypertension, since vascular disease has been demonstrated to have a multiplicative effect on radiation-induced fibrosis, previous transurethral resection of the prostate, and high-dose RT (notably in the periurethral and apical regions) [11,13,76].

Radiation-induced strictures most commonly occur in the bulbomembranous region in patients that elect for primary RT and at the vesicourethral anastomosis (VUA) in patients electing for salvage or neoadjuvant RT. Urethral strictures form when the relative loss of normal cells is over a certain toxicity–specific threshold. Hughes et al. compared membranous urethral strictures in patients with and without radiation treatment for prostate cancer. Nineteen patients had radiation for treatment and fifty-one had no radiation exposure; the post-radiation specimens had a significantly higher collagen density (*p* = 0.01), higher collagen organization (*p* = 0.0014), increased number of spindle cells (*p* = 0.005), and decreased tissue vascularity (*p* = 0.0005), as well as significant differences in the presence of hyalinized fibrosis (*p* = 0.03), vacuolation (*p* = 0.0001), and fat entrapment (*p* = 0.005) when compared to non-irradiated specimens [67]. Strictures can exhibit different histological properties when comparing recurrent strictures to non-recurrent strictures. Recurrent, radiation-induced strictures featured specific characteristics such as paucicellular plaques with a lower number of stromal cells where the stricture was present [77].

## 9. Surgical Pitfalls and Options for Treating Radiation Strictures

In order to create a treatment plan for a patient with a suspected post-RT stricture, a complete workup is necessary including a physical exam, history of prior urological instrumentation, treatments, and incontinence, as well as diagnostic testing such as urodynamic studies and lab tests. Direct visualization of the stricture and assessment of adjacent tissue should be performed, with retrograde urethrogram (RUG) combined with voiding cystourethrogram (VCUG) used if the retrograde scope cannot pass through the stricture. Additional cross-sectional imaging modalities including CT scan and/or MRI may be required to assess associated pathology (i.e., presence and extent of calcified/necrotic prostatic cavity, or urethrosymphyseal or recto-urethral fistula). Treatment options for post-RT urethral stricture are conservative management (clean intermittent catheterization or chronic catheter placement), endoscopic management (dilation, direct visualization internal urethrotomy [DVIU]), and primary reconstructive options, with joint decision making to be performed between physician and patient based on oncologic and performance status, stricture location/length/number, bladder status, and current level of continence [78,79,80].

## 10. Conservative Management

Patients who are not great candidates for surgery or do not want to undergo more invasive procedures can receive some level of relief from conservative management options for stricture disease. Surgery can carry significant side effects such as new onset or exacerbation of urinary incontinence, along with recurrence of stricture, not to mention the risk associated with general anesthesia [81]. Conservative management options include clean intermittent catheterization and endoscopic techniques. A study conducted by Rozanski et al., investigating 91 men with radiation-induced stricture disease treated with endoscopic management or clean intermittent catheterization, demonstrated that 80% of patients maintained stable uroflowmetry values, post-void residual measurements, and serum creatinine levels on conservative management over a median follow up of 15 months. A total of 90% underwent dilation and 44% underwent direct visual internal urethrotomy. More importantly, conservative management did not increase urinary incontinence rates [81].

Additionally, it is important to acknowledge that not all urethral strictures in irradiated patients need to proceed with additional surgical intervention and depend on the balance between impacting bladder emptying and urinary incontinence. This is inherently dependent on an accurate functional and quality of life evaluation to select those patients that would benefit from interventions listed below [81]. It is important to accurately assess these metrics to decide who would benefit, as resulting incontinence can significantly impact quality of life and even operative measures to address resulting incontinence; male sling placement or artificial urinary sphincter patients have previously been demonstrated to have increased rates of complications in this previously irradiated patient population [82]. In this context, it is prudent to acknowledge that reconstructive techniques such as dorsal onlay urethroplasty are associated with incontinence as well with rates of less than 10% to guide patient counseling [83].

## 11. Endoscopic Management

Dilation and DVIU are endoscopic procedures that can be used in initial interventions for stricture disease; however, these measures have limited long-term success in patients with radiation-induced urethral strictures. A study by Merrick et al. revealed that among 29 patients who had undergone endoscopic treatment of their strictures, 31% needed multiple procedures to achieve patency, and 3 of the patients required suprapubic tube placement after developing recurrent strictures [14]. Similar findings from other publications revealed that nearly half of patients treated endoscopically needed subsequent interventions for stricture treatment. Notably, one study highlighted the significant risk of de novo incontinence associated with endoscopic approaches [13].

It should be noted that the location of the stricture and which endoscopic procedure is performed impact the outcome. Pfalzgraf et al. observed that patients with VUAS had a higher likelihood of experiencing de novo incontinence after transurethral incision of the stenosis compared to after transurethral resection (31% vs. 12%, *p* = 0.032). The authors were unable to identify any discernable variables such as history of radiation, previous procedures, or endoscopic procedures that were indicative of success [6,84]. Given the futility of endoscopic incisions or dilations for treating radiation-induced strictures, these techniques may be confined to patients who are unable to tolerate general anesthesia or are resistant to more invasive procedures.

The pursuit of a more durable result following endoscopic management has led to the adjunctive use of injectable substances which may inhibit fibrosis and thus prevent recurrent stenosis. The injection of steroids and mitomycin C following DVIU have demonstrated patency rates of 83% and 90%, respectively [85,86,87]. Although these numbers must be considered in their proper context, as multiple procedures are often needed to achieve them, a sub-analysis of radiated patients shows significantly worse success rates, and mitomycin can have severe adverse events resulting in the need for cystectomy [87,88,89]. More studies will be needed before conclusions regarding the adjuvant injection of antifibrotics can be made.

## 12. Reconstructive Techniques—Excision and Primary Anastomosis (EPA)

EPA for radiation-induced urethral strictures is typically performed via perineal access and tends to be more complicated compared to EPA performed on non-radiated urethral strictures for two main reasons: (1) the significant radiation-induced fibrosis which makes urethral mobilization difficult and (2) radiated fields have poor vascularity resulting in poor wound healing.

The success rate of EPA, defined as not requiring additional procedures, for radiation-induced urethral stricture is reported to be 65–95% [90,91,92,93,94]. A multi-institutional retrospective study by Voelzke et al. identified the following factors, age, stricture length, and EBRT + BT, as associated with stricture recurrence after EPA [95]. One major limitation of this study is the heterogeneity in auxiliary surgical maneuvers (use of gracilis flaps, inferior pubectomy, crural separation) employed to complete the reconstruction, thus the effect of this excisional technique alone on the outcomes is difficult to ascertain.

De-novo incontinence rates have been documented to reach up to 36% with 13–17% needing eventual artificial urinary sphincter placement [90,91,92,96]. A study performed by Chung et al. evaluated continence outcomes in patients with radiation-induced strictures and compared them to a control group of patients with pelvic fracture urethral disruption injury, with both undergoing EPA. Radiated patients exhibited a higher (33%) outcome of de-novo stress incontinence compared to the control group (12%) [94]. These findings indicate that exposure to radiation may serve as a risk factor for developing de-novo incontinence, underlying the importance of surgeons discussing the increased likelihood of urinary incontinence with patients interested in undergoing urethroplasty for radiation-induced stricture disease [95].

## 13. Reconstructive Techniques—Buccal Mucosa Graft Urethroplasty

In the past, augmentation techniques were not performed on radiation patients due to concerns regarding the viability of the graft in such hostile environments. Recent studies suggest that augmented urethroplasties using buccal mucosa grafts (BMG), the most versatile grafts currently used in urologic practice, are a viable repair technique for post-radiation strictures and have outcomes similar to non-radiated patients in the short term and medium term [67]. Buccal mucosa grafts (ventral and dorsal onlays) have been used in several suggested approaches of augmentation urethroplasty which provide patients with outstanding outcomes with regard to postoperative patency rates; they have been documented to be from 71 to 75% and even 100% in one small series [83,92,96]. A study by Ahyai et al. on outcomes following ventral onlay BMG urethroplasty reported an overall success rate was 71% at a mean follow up of 26.5 months, and rates of de novo incontinence and erectile dysfunction were 10.5% and 6.3%, respectively [97].

Blakely et al. documented findings on three patients with membranous urethral strictures who had undergone dorsal onlay BMG urethroplasty after RT. The results showed that all patients were able to maintain patency with no instances of de novo incontinence during the follow up at 8 months [95]. A subsequent multicenter retrospective review by Policastro et al. of 79 patients with posterior urethral stenosis secondary to radiation therapy who underwent dorsal onlay buccal mucosal urethroplasty with a 3 cm mean stricture length demonstrated an 82.3% stricture-free rate at a mean 21 months of follow up, 8% de novo stress urinary incontinence, and 91% patient satisfaction [98]. Three studies have shown that the dorsal onlay technique for radiation strictures for BMG urethroplasty patients has not been associated with worsening erection quality, which is frequent in anastomotic urethroplasty [92,96,99].

Additionally, a single-center retrospective cohort review by Vetterlein et al. reported on 47 patients undergoing ventral onlay buccal mucosal urethroplasty (2009–2016) with a median graft length of 5 cm and demonstrated a recurrence rate of 33%. The authors utilized a validated questionnaire (USS-PROM) showing a mean six-item LUTS score of 7.2 and a mean IIEF-EF score of 4.4. Ultimately, the questionnaire demonstrated that 53% of patients experienced daily urinary incontinence and 26% underwent an artificial urinary sphincter placement. However, in regard to satisfaction, 71% of patients were ultimately satisfied with the outcome [100].

Given the lack of direct comparisons in the literature, further comparative data on repair durability, rates of de novo incontinence, and patient-reported outcomes are required to determine the most suitable technique for managing radiation-induced stricture disease. However, based on the existing literature, surgical reconstructive techniques seem to be viable options for treating stricture disease in such a challenging environment.

## 14. Robotic Techniques

Robotic surgery has grown as a possible alternative to endoscopic management and open surgery in the treatment of radiation-induced urethral strictures. Open surgery for radiation-induced urethral strictures can lead to longer recovery times, additional interventions, and wound complications due to the hampered healing process present in irradiated tissue [101,102]. A review of open post-radiation urologic reconstructive procedures found a morbidity rate up to 54% [100,101]. In comparison, robotic surgery has been linked with reduced early postoperative morbidities for other major urologic procedures. This approach may translate to post-radiation reconstruction, potentially improving outcomes and decreasing morbidities [101,103,104].

Traditionally, open perineal surgery was used for the reconstruction of the posterior urethra, but this technique grew to be more challenging after RT since the natural planes of the tissue are eliminated and fibrosis is present [93,101]. In these circumstances, robotic surgery can improve visualization and improve dexterity in a tight working space around the bladder neck, allowing for the accurate placement of proximal sutures [101,105,106]. In patients undergoing a repair of vesicourethral anastomotic stenosis, robotic reconstruction of posterior urethral stenosis demonstrated a 100% patency rate. However, in two out of the seven patients, artificial urinary sphincter placement was needed [107,108]. A true assessment of the robotic approach for the repair of post-radiation stenosis is limited due to the exclusion of patients with a history of radiation in most of the current literature. In the only assessment to date assessing the feasibility of robotic VUAS repair in radiation patients, an honest accounting of outcomes revealed worse outcomes including longer operative times, higher rates of incontinence, and higher recurrence rates leading to higher rates of reintervention compared to non-radiated counterparts [107]. In a study with six patients who underwent robotic posterior urethral stenosis repair with prior history of RP and half of these patients also underwent salvage RT, Lavollé et al. noted after robotic repair that 50% of the patients required further endoscopic intervention and 50% of the patients developed incontinence that required artificial urinary sphincter placement [107,109].

## 15. Current Trends and Future Directions

Recent developments in surgical techniques, such as non-transection, artery sparing EPA, and intra-sphincter bulboprostatic anastomotic techniques may improve outcomes for radiation-induced urethral stricture disease. The use of BMG for post-radiation stenosis is increasingly accepted as the volume of literature confirming the graft’s ability to survive in a radiated field grows. In that vein, future research is focused on understanding the oral environment during healing to replicate that microenvironment and decrease fibrosis and scar formation [110]. In order to increase healing without hypertrophic scar formation and to lower infection, using BMG in combination with non-destructive surgical techniques may be the best option.

Patients in this population can either be managed conservatively or offered formal reconstruction, depending on patient comorbidities and preference. However, direct comparison studies between excision and augmented techniques are still needed to determine if there is a difference in recurrence, erectile function, and incontinence.

Radiation can activate certain proteomic and biochemical changes that contribute to the creation of strictures. Future research will be imperative to better understand these changes, specifically regarding the effect of various RT modalities on the bladder neck and urethra. This will help forecast the results of reconstructive surgery alongside developing a greater understanding and measuring of the mechanisms behind the damage to the urethra. Pre-treatment using prime innate DNA repair mechanisms to avoid radiation-induced changes or molecules that can sequester reactive oxygen species (ROS) require further study of the pathophysiology behind the effects of radiation on tissue, although some possible avenues of investigation include pre-treatment with an ROS scavenger, tyrosine kinase inhibitors, and hyperbaric oxygen therapy [111,112,113]. There are ongoing investigations regarding stem cell therapy to stop or undo the tissue damage from radiation. These interventions include platelet-rich plasma, fat grafting, umbilical cord blood, and injection of adipose stem cells [114,115,116,117,118].

## 16. Limitations of Study

Urethroplasty has demonstrated itself as an effective method of dealing with radiation-induced urethral stricture, as shown in Sapienza et al., where strictures were removed successfully 80% of the time, showing a similar efficacy to the 85% success rate in tissue that was not irradiated [119,120]. In order to determine more effective recommendations for clinicians, more head-to-head comparison studies are needed, especially with regards to buccal mucosa graft urethroplasty and EPA to establish long term changes, noting the advantages and disadvantages of each procedure.

Studies included patients that have undergone BT, EBRT, and sometimes combination therapy, analyzing the rate of stricture formation associated with each modality. However, it was not delineated if each modality of RT lead to more recalcitrant strictures. These data would be able to better inform clinicians when counseling their patients regarding RT.

Previous studies on robotic techniques in stricture repair have excluded patients with prior RT. The current literature studying robotic stricture repair in radiation patients has smaller sample sizes and will be worth exploring further, given the efficacy of robotic techniques for other urological conditions.

## 17. Conclusions

Radiation-induced urethral stricture disease is a prevalent complication of prostatic radiation through imbalances of pro- and anti-apoptotic signaling mechanisms. Conservative management options (intermittent catheterization or endoscopic options) have poor durability but are effective options for patients that do not want to have a procedure under general anesthesia. EPA and augmented repairs have similar rates of recurrence, but augmented repairs seem to have lower rates of de novo urinary incontinence. However, head-to-head comparisons are needed to confirm this. Buccal mucosa has been shown to be a hardy tissue that can survive an irradiated tissue environment, and there is a growing body of literature reporting its viability in urethroplasty. However, future studies including randomized control trials and head-to-head comparisons will be required. Being able to address the damage from radiation immediately through pre-treatment can offer a proactive approach to reduce the need for complex procedures. Finally, more research is needed to understand the changes induced by radiation, how they result in stricture formation, and what may be done to reverse these changes.

## Figures and Tables

**Table 1:** Scale of severity for inflammation, fibrosis, vascularity, and cellular alterations to evaluate radio-induced injury, as proposed by Gallet et al. [58].

Score		Grade 0	Grade 1	Grade 2	Grade 3
Inflammation Score	Skin	No inflammatory infiltrate <2 mast cells	Some inflammatory elements and mast cells	Frequent inflammatory infiltrates	Ubiquitary inflammatory infiltrates
	Muscle	No inflammatory elements	Some inflammatory elements	Frequent inflammatory infiltrates and mast cells	Ubiquitary inflammatory infiltrates
Fibrosis Score	Skin	Normal structure	Minor fibrosis with preserved structure	Moderate fibrosis, modification of structure	Major fibrosis, destructuration
	Muscle	Normal structure	Fibrosis equal or <10% of the slide	Fibrosis, equal or <20% of the slide	Fibrosis, >20% of the slide
Vascular Score	Vessels	Normal number and aspect of vessels	Diminution of number of vessels and/or moderate vascular damage	Rare vessels and/or moderate vascular damage	No vessels or major alterations (thrombosis, endothelial damage, or diffuse + staining pattern)
Cellular Alterations Score	Skin	Normal epithelium, presence of fibroblasts and hair cells	Minor thickening of epithelium, rarefaction of fibroblasts, myofibroblasts, some dystrophic nuclei	Moderate thickening of epithelium, necrosis of epithelial cells, rarefaction of fibroblasts, myofibroblasts, frequent dystrophic nuclei	Important thickening of epithelium, frequent necrosis of epithelial cells, rarefaction of fibroblasts, myofibroblasts, dystrophic nuclei
	Muscle	Normal	Some dystrophic nuclei	Dystrophic nuclei, rare aspects of fibrosis	Dystrophic nuclei, fibrosis

## Data Availability

Full data will be provided by the main investigator upon reasonable request.

## References

[1] CDC US Cancer Statistics 2019. cdc.gov/USCS.

[2] Tang C., Hoffman K.E., Allen P.K., Gabel M., Schreiber D., Choi S., Chapin B.F., Nguyen Q., Davis J.W., Corn P. (2019). Contemporary prostate cancer treatment choices in multidisciplinary clinics referenced to national trends. Cancer.

[3] Del Giudice F., Huang J., Li S., Sorensen S., Enemchukwu E., Maggi M., Salciccia S., Ferro M., Crocetto F., Pandolfo S.D. (2022). Contemporary trends in the surgical management of urinary incontinence after radical prostatectomy in the United States. Prostate Cancer Prostatic Dis..

[4] Boorjian S.A., Eastham J.A., Graefen M., Guillonneau B., Karnes R.J., Moul J.W., Schaeffer E.M., Stief C., Zorn K.C. (2012). A Critical Analysis of the Long-Term Impact of Radical Prostatectomy on Cancer Control and Function Outcomes. Eur. Urol..

[5] Elliott S.P., Meng M.V., Elkin E.P., McAninch J.W., Duchane J., Carroll P.R., CaPSURE Investigators (2007). Incidence of urethral stricture after primary treatment for prostate cancer: Data from CaPSURE. J. Urol..

[6] Sterling J., Policastro C., Nikolavsky D. (2022). Pathophysiology of radiation-induced urethral strictures and therapeutic strategies optimizing outcomes of surgical repair. Scientific Advances in Reconstructive Urology and Tissue Engineering.

[7] Liberman D., Mehus B., Elliott S.P. (2014). Urinary adverse effects of pelvic radiotherapy. Transl. Androl. Urol..

[8] David R.V., Kahokehr A.A., Lee J., Watson D.I., Leung J., O’callaghan M.E. (2022). Incidence of genitourinary complications following radiation therapy for localised prostate cancer. World J. Urol..

[9] Muise A., Pan M.M., Rose B., Buckley J.C. (2023). Functional outcomes after prostate cancer treatment: A comparison between single and multiple modalities. Urol. Oncol. Semin. Orig. Investig..

[10] Ma J.L., Hennessey D.B., Newell B.P., Bolton D.M., Lawrentschuk N. (2018). Radiotherapy-related complications presenting to a urology department: A more common problem than previously thought?. BJU Int..

[11] Hindson B.R., Millar J.L., Matheson B. (2012). Urethral strictures following high-dose-rate brachytherapy for prostate cancer: Analysis of risk factors. Brachytherapy.

[12] Awad M.A., Gaither T.W., Osterberg E.C., Murphy G.P., Baradaran N., Breyer B.N. (2018). Prostate cancer radiation and urethral strictures: A systematic review and meta-analysis. Prostate Cancer Prostatic Dis..

[13] Sullivan L., Williams S.G., Tai K.H., Foroudi F., Cleeve L., Duchesne G. (2009). Urethral stricture following high dose rate brachytherapy for prostate cancer. Radiother. Oncol..

[14] Merrick G.S., Butler W.M., Wallner K.E., Galbreath R.W., Anderson R.L., Allen Z.A., Adamovich E. (2006). Risk Factors for the Development of Prostate Brachytherapy Related Urethral Strictures. J. Urol..

[15] Marks L.B., Carroll P.R., Dugan T.C., Anscher M.S. (1995). The response of the urinary bladder, urethra, and ureter to radiation and chemotherapy. Int. J. Radiat. Oncol..

[16] Sowerby R.J., Gani J., Yim H., Radomski S.B., Catton C. (2014). Long-term complications in men who have early or late radio-therapy after radical prostatectomy. Can. Urol. Assoc. J..

[17] Cavalcanti A.G., Yucel S., Deng D.Y., McANINCH J.W., Baskin L.S. (2004). The Distribution of Neuronal and Inducible Nitric Oxide Synthase in Urethral Stricture Formation. J. Urol..

[18] Simsek A., Aldamanhori R., Chapple C.R., MacNeil S. (2018). Overcoming scarring in the urethra: Challenges for tissue engineering. Asian J. Urol..

[19] Mundy A.R., Andrich D.E. (2011). Urethral strictures. BJU Int..

[20] Hofer M.D., Cheng E.Y., Bury M.I., Park E., Xu W., Hong S.J., Kaplan W.E., Sharma A.K. (2014). Analysis of Primary Urethral Wound Healing in the Rat. Urology.

[21] Singh M., Blandy J. (1976). The Pathology of Urethral Stricture. J. Urol..

[22] Baskin L.S., Constantinescu S.C., Howard P.S., McAninch J.W., Ewalt D.H., Duckett J.W., Snyder H.M., Macarak E.J. (1993). Biochemical Characterization and Quantitation of the Collagenous Components of Urethral Stricture Tissue. J. Urol..

[23] Brock G., Nunes L., Padma-Nathan H., Boyd S., Lue T.F. (1993). Nitric oxide synthase: A new diagnostic tool for neurogenic impotence. Urology.

[24] Da-Silva E.A., Sampaio F.J., Dornas M.C., Damiao R., Cardoso L.E. (2002). Extracellular matrix changes in urethral stricture disease. J. Urol..

[25] Stoddard N., Leslie S.W. (2021). Histology, Male Urethra.

[26] Rodemann H.P., Blaese M.A. (2007). Responses of Normal Cells to Ionizing Radiation. Semin. Radiat. Oncol..

[27] Fajardo L.F. (2005). The pathology of ionizing radiation as defined by morphologic patterns. Acta Oncol..

[28] Fajardo L.F. (1999). Is the pathology of radiation injury different in small vs large blood vessels?. Cardiovasc. Radiat. Med..

[29] Peña L.A., Fuks Z., Kolesnick R.N. (2000). Radiation-induced apoptosis of endothelial cells in the murine central nervous system: Protection by fibroblast growth factor and sphingomyelinase deficiency. Cancer Res..

[30] Lin T., Genestier L., Pinkoski M.J., Castro A., Nicholas S., Mogil R., Paris F., Fuks Z., Schuchman E.H., Kolesnick R.N. (2000). Role of Acidic Sphingomyelinase in Fas/CD95-mediated Cell Death. J. Biol. Chem..

[31] Reyes J.G., Robayna I.G., Delgado P.S., González I.H., Aguiar J.Q., Rosas F.E., Fanjul L.F., de Galarreta C.M.R. (1996). c-Jun Is a Downstream Target for Ceramide-activated Protein Phosphatase in A431 Cells. J. Biol. Chem..

[32] Dressler K.A., Mathias S., Kolesnick R.N. (1992). Tumor Necrosis Factor-α Activates the Sphingomyelin Signal Transduction Pathway in a Cell-Free System. Science.

[33] Müller G., Ayoub M., Storz P., Rennecke J., Fabbro D., Pfizenmaier K. (1995). PKC zeta is a molecular switch in signal transduction of TNF-alpha, bifunctionally regulated by ceramide and arachidonic acid. EMBO J..

[34] Haimovitz-Friedman A., Kan C.C., Ehleiter D., Persaud R.S., McLoughlin M., Fuks Z., Kolesnick R.N. (1994). Ionizing radiation acts on cellular membranes to generate ceramide and initiate apoptosis. J. Exp. Med..

[35] Haimovitz-Friedman A., Balaban N., McLoughlin M., Ehleiter D., Michaeli J., Vlodavsky I., Fuks Z. (1994). Protein kinase C mediates basic fibroblast growth factor protection of endothelial cells against radiation-induced apoptosis. Cancer Res..

[36] Verheij M., Bose R., Lin X.H., Yao B., Jarvis W.D., Grant S., Birrer M.J., Szabo E., Zon L.I., Kyriakis J.M. (1996). Requirement for ceramide-initiated SAPK/JNK signalling in stress-induced apoptosis. Nature.

[37] Basu S., Bayoumy S., Zhang Y., Lozano J., Kolesnick R. (1998). BAD Enables Ceramide to Signal Apoptosis via Ras and Raf-1. J. Biol. Chem..

[38] Yang J., Liu X., Bhalla K., Kim C.N., Ibrado A.M., Cai J., Peng T.-I., Jones D.P., Wang X. (1997). Prevention of Apoptosis by Bcl-2: Release of Cytochrome c from Mitochondria Blocked. Science.

[39] Belka C., Budach W. (2002). Anti-apoptotic Bcl-2 proteins: Structure, function and relevance for radiation biology. Int. J. Radiat. Biol..

[40] Stroh C., Schulze-Osthoff K. (1998). Death by a thousand cuts: An ever increasing list of caspase substrates. Cell Death Differ..

[41] Canman C.E., Kastan M.B. (1997). Role of p53 in Apoptosis. Adv. Pharmacol..

[42] Herr I., Debatin K.-M. (2001). Cellular stress response and apoptosis in cancer therapy. Blood.

[43] Tan J., Geng L., Yazlovitskaya E.M., Hallahan D.E. (2006). Protein kinase B/Akt-dependent phosphorylation of glycogen synthase kinase-3beta in irradiated vascular endothelium. Cancer Res..

[44] Toulany M., Kasten-Pisula U., Brammer I., Wang S., Chen J., Dittmann K., Baumann M., Dikomey E., Rodemann H.P. (2006). Blockage of Epidermal Growth Factor Receptor-Phosphatidylinositol 3-Kinase-AKT Signaling Increases Radiosensitivity of K-*RAS* Mutated Human Tumor Cells In vitro by Affecting DNA Repair. Clin. Cancer Res..

[45] Bayreuther K., Francz P., Rodemann H. (1992). Fibroblasts in normal and pathological terminal differentiation, aging, apoptosis and transformation. Arch. Gerontol. Geriatr..

[46] Rodemann H.P., Binder A., Burger A., Güven N., Löffler H., Bamberg M. (1996). The underlying cellular mechanism of fibrosis. Kidney Int. Suppl..

[47] Burger A., Löffler H., Bamberg M., Rodemann H.P. (1998). Molecular and cellular basis of radiation fibrosis. Int. J. Radiat. Biol..

[48] Rodemann H., Bamberg M. (1995). Cellular basis of radiation-induced fibrosis. Radiother. Oncol..

[49] Paun A., Kunwar A., Haston C.K. (2015). Acute adaptive immune response correlates with late radiation-induced pulmonary fibrosis in mice. Radiat. Oncol..

[50] Takigawa N., Segawa Y., Saeki T., Kataoka M., Ida M., Kishino D., Fujiwara K., Ohsumi S., Eguchi K., Takashima S. (2000). Bronchiolitis obliterans organizing pneumonia syndrome in breast-conserving therapy for early breast cancer: Radiation-induced lung toxicity. Int. J. Radiat. Oncol. Biol. Phys..

[51] Bessout R., Demarquay C., Moussa L., René A., Doix B., Benderitter M., Sémont A., Mathieu N. (2015). TH17 predominant T-cell responses in radiation-induced bowel disease are modulated by treatment with adipose-derived mesenchymal stromal cells. J. Pathol..

[52] Garrido-Mesa N., Algieri F., Rodriguez Nogales A., Galvez J. (2013). Functional plasticity of Th17 cells: Implications in gastrointestinal tract function. Int. Rev. Immunol..

[53] Mesquita D., Cruvinel W.D.M., Câmara N.O.S., Kállas E.G., Andrade L.E.C. (2009). Autoimmune diseases in the TH17 era. Braz. J. Med. Biol. Res..

[54] Barron L., Wynn T.A. (2011). Fibrosis is regulated by Th2 and Th17 responses and by dynamic interactions between fibroblasts and macrophages. Am. J. Physiol. Liver Physiol..

[55] Martin M., Lefaix J., Delanian S. (2000). TGF-beta1 and radiation fibrosis: A master switch and a specific therapeutic target?. Int. J. Radiat. Oncol. Biol. Phys..

[56] Mayer M. (1990). Biochemical and biological aspects of the plasminogen activation system. Clin. Biochem..

[57] Hageman J., Eggen B.J., Rozema T., Damman K., Kampinga H.H., Coppes R.P. (2005). Radiation and transforming growth factor-beta cooperate in transcriptional activation of the profibrotic plasminogen activator inhibitor-1 gene. Clin. Cancer Res..

[58] Gallet P., Phulpin B., Merlin J.L., Leroux A., Bravetti P., Mecellem H., Tran N., Dolivet G. (2011). Long-term alterations of cy-tokines and growth factors expression in irradiated tissues and relation with histological severity scoring. PLoS ONE.

[59] Hall C.R., Ehrenpreis E.D., Marsh R.D.W., Small W. (2015). Pathology of radiation effects on healthy tissues in the pelvis. Radiation Therapy for Pelvic Malignancy and Its Consequences.

[60] Fajardo L.F., Berthrong M. (1978). Radiation injury in surgical pathology. Am. J. Surg. Pathol..

[61] Antonakopoulos G.N., Hicks R.M., Hamilton E., Berry R.J. (1982). Early and late morphological changes (including carcinoma of the urothelium) induced by irradiation of the rat urinary bladder. Br. J. Cancer.

[62] Zuppone S., Bresolin A., Spinelli A.E., Fallara G., Lucianò R., Scarfò F., Benigni F., Di Muzio N., Fiorino C., Briganti A. (2020). Pre-clinical Research on Bladder Toxicity After Radiotherapy for Pelvic Cancers: State-of-the Art and Challenges. Front. Oncol..

[63] Jaal J., Brüchner K., Hoinkis C., Dörr W., Doerr W. (2004). Radiation-induced variations in urothelial expression of intercellular adhesion molecule 1 (ICAM-1): Association with changes in urinary bladder function. Int. J. Radiat. Biol..

[64] Kraft M., Oussoren Y., Stewart F.A., Dörr W., Schultz-Hector S. (1996). Radiation-induced changes in transforming growth factor beta and collagen expression in the murine bladder wall and its correlation with bladder function. Radiat. Res..

[65] Carrier S., Hricak H., Lee S.S., Baba K., Morgan D.M., Nunes L., Ross G.Y., Phillips T.L., Lue T.F. (1995). Radiation-induced decrease in nitric oxide synthase--containing nerves in the rat penis. Radiology.

[66] Van Der Wielen G.J., Vermeij M., De Jong B.W., Schuit M., Marijnissen J., Kok D.J., Van Weerden W.M., Incrocci L. (2009). Changes in the Penile Arteries of the Rat after Fractionated Irradiation of the Prostate: A Pilot Study. J. Sex. Med..

[67] Hughes M., Caza T., Li G., Daugherty M., Blakley S., Nikolavsky D. (2019). Histologic characterization of the post-radiation urethral stenosis in men treated for prostate cancer. World J. Urol..

[68] Hanin L., Zaider M. (2013). A mechanistic description of radiation-induced damage to normal tissue and its healing kinetics. Phys. Med. Biol..

[69] Martin J.M., Richardson M., Siva S., Cardoso M., Handmer M., Sidhom M. (2022). Mechanisms, mitigation, and management of urinary toxicity from prostate radiotherapy. Lancet Oncol..

[70] Rodda S., Tyldesley S., Morris W.J., Keyes M., Halperin R., Pai H., McKenzie M., Duncan G., Morton G., Hamm J. (2017). ASCENDE-RT: An Analysis of Treatment-Related Morbidity for a Randomized Trial Comparing a Low-Dose-Rate Brachytherapy Boost with a Dose-Escalated External Beam Boost for High- and Intermediate-Risk Prostate Cancer. Int. J. Radiat. Oncol..

[71] Herschorn S., Elliott S., Coburn M., Wessells H., Zinman L. (2014). SIU/ICUD Consultation on Urethral Strictures: Posterior Urethral Stenosis After Treatment of Prostate Cancer. Urology.

[72] Merrick G.S., Butler W.M., Wallner K.E., Galbreath R.W., Lief J.H. (2003). Long-term urinary quality of life after permanent prostate brachytherapy. Int. J. Radiat. Oncol..

[73] Allen Z.A., Merrick G.S., Butler W.M., Wallner K.E., Kurko B., Anderson R.L., Murray B.C., Galbreath R.W. (2005). Detailed urethral dosimetry in the evaluation of prostate brachytherapy-related urinary morbidity. Int. J. Radiat. Oncol..

[74] Zaffuto E., Gandaglia G., Fossati N., Dell’Oglio P., Moschini M., Cucchiara V., Suardi N., Mirone V., Bandini M., Shariat S.F. (2017). Early Postoperative radiotherapy is assocciated with worse functional outcomes in patients with prostate cancer. J. Urol..

[75] Tibbs M.K. (1997). Wound healing following radiation therapy: A review. Radiother. Oncol..

[76] Campos-Juanatey F., Martín J.P., Illanes R.G., Ramos L.V. (2017). Nontraumatic posterior urethral stenosis. Actas Urol. Esp..

[77] Samarska I.V., Dani H., Bivalacqua T.J., Burnett A.L., Matoso A. (2021). Histopathologic and clinical comparison of recurrent and non-recurrent urethral stricture disease treated by reconstructive surgery. Transl. Androl. Urol..

[78] Barry J.M. (1989). Visual urethrotomy in the management of the obliterated membranous urethra. Urol. Clin. N. Am..

[79] Stein D.M., Santucci R.A. (2015). Pro: Endoscopic realignment for pelvic fracture urethral injuries. Transl. Androl. Urol..

[80] Yasuda K., Yamanishi T., Isaka S., Okano T., Masai M., Shimazaki J. (1991). Endoscopic Re-Establishment of Membranous Urethral Disruption. J. Urol..

[81] Rozanski A.T., Moynihan M.J., Zhang L.T., Muise A.C., Holst D.D., Copacino S.A., Zinman L.N., Buckley J.C., Vanni A.J. (2022). The Efficacy and Safety of a Conservative Management Approach to Radiation-Induced Male Urethral Strictures in Elderly Patients With Comorbidities. Société Int. d’Urologie J..

[82] Ravier E., Fassi-Fehri H., Crouzet S., Gelet A., Abid N., Martin X. (2014). Complications after artificial urinary sphincter implantation in patients with or without prior radiotherapy. BJU Int..

[83] Blakely S., Caza T., Landas S., Nikolavsky D. (2015). Dorsal Onlay Urethroplasty for Membranous Urethral Strictures: Urinary and Erectile Functional Outcomes. J. Urol..

[84] Pfalzgraf D., Worst T., Kranz J., Steffens J., Salomon G., Fisch M., Reiß C.P., Vetterlein M.W., Rosenbaum C.M. (2021). Vesico-urethral anastomotic stenosis following radical prostatectomy: A multi-institutional outcome analysis with a focus on en-doscopic approach, surgical sequence, and the impact of radiation therapy. World J. Urol..

[85] Eltahawy E., Gur U., Virasoro R., Schlossberg S.M., Jordan G.H. (2008). Management of recurrent anastomotic stenosis following radical prostatectomy using holmium laser and steroid injection. BJU Int..

[86] Kravchick S., Lobik L., Peled R., Cytron S. (2013). Transrectal Ultrasonography-Guided Injection of Long-Acting Steroids in the Treatment of Recurrent/Resistant Anastomotic Stenosis After Radical Prostatectomy. J. Endourol..

[87] Redshaw J.D., Broghammer J.A., Smith T.G., Voelzke B.B., Erickson B.A., McClung C.D., Elliott S.P., Alsikafi N.F., Presson A.P., Aberger M.E. (2015). Intralesional injection of mitomycin-C at transurethral incision of bladder neck contracture may offer limited benefit: TURNS Study Group. J. Urol..

[88] Rozanski A.T., Zhang L.T., Holst D.D., Copacino S.A., Vanni A.J., Buckley J.C. (2020). The Effect of Radiation Therapy on the Efficacy of Internal Urethrotomy With Intralesional Mitomycin C for Recurrent Vesicourethral Anastomotic Stenoses and Bladder Neck Contractures: A Multi-Institutional Experience. Urology.

[89] Vanni A.J., Zinman L.N., Buckley J.C. (2011). Radial Urethrotomy and Intralesional Mitomycin C for the Management of Recurrent Bladder Neck Contractures. J. Urol..

[90] Meeks J.J., Brandes S.B., Morey A.F., Thom M., Mehdiratta N., Valadez C., Granieri M.A., Gonzalez C.M. (2011). Urethroplasty for Radiotherapy Induced Bulbomembranous Strictures: A Multi-Institutional Experience. J. Urol..

[91] Hofer M.D., Zhao L.C., Morey A.F., Scott J.F., Chang A.J., Brandes S.B., Gonzalez C.M. (2014). Outcomes after Urethroplasty for Radiotherapy Induced Bulbomembranous Urethral Stricture Disease. J. Urol..

[92] Rourke K., Kinnaird A., Zorn J. (2015). Observations and outcomes of urethroplasty for bulbomembranous stenosis after radiation therapy for prostate cancer. World J. Urol..

[93] Fuchs J.S., Hofer M.D., Sheth K.R., Cordon B.H., Scott J.M., Morey A.F. (2016). Improving Outcomes of Bulbomembranous Urethroplasty for Radiation-induced Urethral Strictures in Post-Urolume Era. Urology.

[94] Glass A.S., McAninch J.W., Zaid U.B., Cinman N.M., Breyer B.N. (2012). Urethroplasty After Radiation Therapy for Prostate Cancer. Urology.

[95] Voelzke B.B., Leddy L.S., Myers J.B., Breyer B.N., Alsikafi N.F., Broghammer J.A., Elliott S.P., Vanni A.J., Erickson B.A., Buckley J.C. (2021). Multi-institutional outcomes and associations after excision and primary anastomosis for radiotherapy-associated bulbomembranous urethral stenoses following prostate cancer treatment. Urology.

[96] Chung P.H., Esposito P., Wessells H., Voelzke B.B. (2018). Incidence of Stress Urinary Incontinence After Posterior Urethroplasty for Radiation-induced Urethral Strictures. Urology.

[97] Ahyai S.A., Schmid M., Kuhl M., Kluth L.A., Soave A., Riechardt S., Chun F.K.-H., Engel O., Fisch M., Dahlem R. (2015). Outcomes of Ventral Onlay Buccal Mucosa Graft Urethroplasty in Patients after Radiotherapy. J. Urol..

[98] Policastro C.G., Simhan J., Martins F.E., Lumen N., Venkatesan K., Angulo J.C., Gupta S., Rusilko P., Pérez E.A.R., Redger K. (2020). A multi-institutional critical assessment of dorsal onlay urethroplasty for post-radiation urethral stenosis. World J. Urol..

[99] Virasoro R., Zuckerman J.M., McCammon K.A., Delong J.M., Tonkin J.B., Capiel L., Rovegno A.R., Favre G., Giudice C.R., Eltahawy E.A. (2015). International multi-institutional experience with the vessel-sparing technique to reconstruct the proximal bulbar urethra: Mid-term results. World J. Urol..

[100] Vetterlein M.W., Kluth L.A., Zumstein V., Meyer C.P., Ludwig T.A., Soave A., Riechardt S., Engel O., Dahlem R., Fisch M. (2020). Buccal mucosal graft urethroplasty for radiation-induced urethral strictures: An evaluation using the extended Urethral Stricture Surgery Patient-Reported Outcome Measure (USS PROM). World J. Urol..

[101] Elbakry A.A., Pan M.M., Buckley J.C. (2022). Frontiers in post-radiation urologic reconstruction; robotic surgery and near-infrared fluorescence imaging: A Narrative Review. AME Med. J..

[102] Toia B., Seth J., Ecclestone H., Pakzad M., Hamid R., Greenwell T., Ockrim J. (2019). Outcomes of reconstructive urinary tract surgery after pelvic radiotherapy. Scand. J. Urol..

[103] Flamiatos J.F., Chen Y., Lambert W.E., Martinez Acevedo A., Becker T.M., Bash J.C., Amling C.L. (2019). Open versus robot-assisted radical cystectomy: 30-day perioperative comparison and predictors for cost-to patient, complication, and read-mission. J. Robot. Surg..

[104] Khalil M.I., Tourchi A., Langford B.T., Bhandari N.R., Payakachat N., Davis R., Safaan A., Raheem O.A., Kamel M.H. (2020). Early Postoperative Morbidity of Robotic Versus Open Radical Cystectomy in Obese Patients. J. Endourol..

[105] Kim S., Buckley J.C. (2020). Robotic Lower Urinary Tract Reconstruction. Urol. Clin. N. Am..

[106] Unterberg S.H., Patel S.H., Fuller T.W., Buckley J.C. (2019). Robotic assisted proximal perineal urethroplasty: Improving visualization and ergonomics. Urology.

[107] Bearrick E.N., Findlay B.L., Maciejko L.A., Hebert K.J., Anderson K.T., Viers B.R. (2022). Robotic urethral reconstruction out-comes in men with posterior urethral stenosis. Urology.

[108] Kirshenbaum E.J., Zhao L.C., Myers J.B., Elliott S.P., Vanni A.J., Baradaran N., Erickson B.A., Buckley J.C., Voelzke B.B., Granieri M.A. (2018). Patency and Incontinence Rates After Robotic Bladder Neck Reconstruction for Vesicourethral Anastomotic Stenosis and Recalcitrant Bladder Neck Contractures: The Trauma and Urologic Reconstructive Network of Surgeons Experience. Urology.

[109] Lavollé A., de la Taille A., Chahwan C., Champy C.M., Grinholtz D., Hoznek A., Yiou R., Vordos D., Ingels A. (2019). Extra-peritoneal robot-assisted vesicourethral reconstruction to manage anastomotic stricture following radical prostatectomy. Urology.

[110] Larjava H., Wiebe C., Gallant-Behm C., Hart D.A., Heino J., Häkkinen L. (2011). Exploring scarless healing of oral soft tissues. J. Can. Dent. Assoc..

[111] Chatterjee A., Kosmacek E.A., Oberley-Deegan R.E. (2017). MnTE-2-PyP treatment, or NOX4 inhibition, protects against radiation-induced damage in mouse primary prostate fibroblasts by inhibiting the TGF-Beta 1 signaling pathway. Radiat. Res..

[112] Affandi T., Ohm A.M., Gaillard D., Haas A., Reyland M.E. (2021). Tyrosine kinase inhibitors protect the salivary gland from radiation damage by increasing DNA double-strand break repair. J. Biol. Chem..

[113] Borab Z., Mirmanesh M.D., Gantz M., Cusano A., Pu L.L. (2017). Systematic review of hyperbaric oxygen therapy for the treatment of radiation-induced skin necrosis. J. Plast. Reconstr. Aesthetic Surg..

[114] Ni X., Sun W., Sun S., Yu J., Wang J., Nie B., Sun Z., Ni X., Cai L., Cao X. (2014). Therapeutic Potential of Adipose Stem Cells in Tissue Repair of Irradiated Skeletal Muscle in a Rabbit Model. Cell. Reprogram..

[115] Borrelli M.R., Deleon N.M.D., Adem S., Patel R.A., Mascharak S., Shen A.H., Irizarry D., Nguyen D., Momeni A., Longaker M.T. (2019). Fat grafting rescues radiation-induced joint contracture. Stem Cells.

[116] Lee J., Jang H., Park S., Myung H., Kim K., Kim H., Jang W.-S., Lee S.-J., Myung J.K., Shim S. (2019). Platelet-rich plasma activates AKT signaling to promote wound healing in a mouse model of radiation-induced skin injury. J. Transl. Med..

[117] Lee C., Shim S., Jang H., Myung H., Lee J., Bae C.H., Myung J.K., Kim M.J., Lee S.B., Jang W.S. (2017). Human umbilical cord blood-derived mesenchymal stromal cells and small intestinal submucosa hydrogel composite promotes combined radia-tion-wound healing of mice. Cytotherapy.

[118] Liu B., Ding F.-X., Liu Y., Xiong G., Lin T., He D.-W., Zhang Y.-Y., Zhang D.-Y., Wei G.-H. (2018). Human umbilical cord-derived mesenchymal stem cells conditioned medium attenuate interstitial fibrosis and stimulate the repair of tubular epithelial cells in an irreversible model of unilateral ureteral obstruction. Nephrology.

[119] Sapienza L.G., Ning M.S., Carvalho E.D.F., Spratt D., Calsavara V.F., McLaughlin P.W., Gomes M.J.L., Baiocchi G., Abu-Isa E. (2021). Efficacy and Incontinence Rates After Urethroplasty for Radiation-induced Urethral Stenosis: A Systematic Review and Meta-analysis. Urology.

[120] Meeks J.J., Erickson B., Granieri M.A., Gonzalez C.M. (2009). Stricture Recurrence After Urethroplasty: A Systematic Review. J. Urol..

